# Nonlinear Tactile Estimation Model Based on Perceptibility of Mechanoreceptors Improves Quantitative Tactile Sensing

**DOI:** 10.3390/s22176697

**Published:** 2022-09-04

**Authors:** Momoko Sagara, Lisako Nobuyama, Kenjiro Takemura

**Affiliations:** 1 Graduate School of Science for Open and Environmental Systems, Keio University, Yokohama 223-8522, Japan; 2 Department of Mechanical Engineering, Keio University, Yokohama 223-8522, Japan

**Keywords:** tactile estimation, nonlinear regression, sensory evaluation, mechanoreceptors, tactile sensor

## Abstract

Tactile sensing has attracted significant attention as a tactile quantitative evaluation method because the tactile sensation is an important factor while evaluating consumer products. Although the human tactile perception mechanism has nonlinearity, previous studies have often developed linear regression models. In contrast, this study proposes a nonlinear tactile estimation model that can estimate sensory evaluation scores from physical measurements. We extracted features from the vibration data obtained by a tactile sensor based on the perceptibility of mechanoreceptors. In parallel, a sensory evaluation test was conducted using 10 evaluation words. Then, the relationship between the extracted features and the tactile evaluation results was modeled using linear/nonlinear regressions. The best model was concluded by comparing the mean squared error between the model predictions and the actual values. The results imply that there are multiple evaluation words suitable for adopting nonlinear regression models, and the average error was 43.8% smaller than that of building only linear regression models.

## 1. Introduction

Since tactile sensation is one of the most important factors when evaluating a consumer product [1,2,3,4], quantitative evaluation methods for tactile sensation are in high demand in product development. In general, a sensory evaluation test is employed to quantify tactile sensations; however, it requires many subjects to participate in a survey, which is costly and time-consuming. Another issue is that even with the same subject, the results may vary depending on differences in the evaluation environment and physical condition. As an alternative to sensory evaluation tests, tactile estimation using physically acquired quantitative data, or tactile sensing, has attracted significant attention.

There are two main purposes for using sensory evaluation. The first is the quality control of the product. Since the surface of products should always have a similar texture as it is desired, a classification model may be used to classify the surface textures from the data acquired by the sensor. The other is to understand what kind of tactile sensation the surface of the manufactured product evokes in people at the product design phase, which is a more upstream process. To evaluate the tactile sensation of products, sensory evaluation by a large number of subjects is necessary for generalization, and the cost is prohibitive. The realization of a regression model that accurately estimates tactile evaluation scores by humans from sensor information contributes to the design of low-cost, high-quality products. The classification is a simple task compared to a regression task and has already been addressed in previous studies. On the other hand, few accurate models for estimating human tactile evaluation scores have been reported.

Some previous studies about tactile sensing have focused on developing tactile sensors [5,6,7,8,9,10,11,12,13,14,15]. Fishel et al. [5] developed a tactile sensing finger, BioTac, which can detect force, vibration, and heat transfer. Lin et al. [6] developed a human skin-inspired, piezoelectric, flexible, multifunctional tactile sensor that can detect and distinguish the magnitudes, positions, and modes of diverse external stimuli, including by slipping, touching, and bending the tactile sensor. Zheng et al. [7] developed a magnetostrictive tactile sensor based on a Galfenol cantilever. The surface properties, including roughness and slipperiness of an object, can be obtained when the sensor slides on an object’s surface. Most tactile sensor development approaches are based on a robotic strategy, which pays little attention to human perceptibility.

Another approach is to extract meaningful information from vibration data obtained by tracing a simple vibration sensor on an object [16,17,18,19,20,21,22,23,24,25,26,27,28,29,30]. Because the four types of mechanoreceptors in our body have different frequency characteristics [31,32,33], they work as filters for vibration stimulations on the skin. This means that the meaningful information for humans is not the entire vibration data, but is hidden in the vibration data. Previous research [22,23,24,25,26,27,28,29,30] focused on extracting this meaningful information; we believe that this method is quite promising because it uses features focused on the characteristics of mechanoreceptors and can contribute to the elucidation of the human tactile perception mechanisms through an engineering approach. These methods include the use of the neuromorphic encoding [22,23,24,25] and hand-made features [26,27,28,29,30]. As an example of the former method, Gupta et al. [22] converted the analog vibration data acquired by the fabricated sensor into a spike train using a neural model and encoded the spatiotemporal activation patterns of mechanoreceptors through a gray-level co-occurrence matrix. They showed that the feature is effective in texture classification and robust to changes in tactile velocity. Yi et al. [23] showed that spike train similarity comparisons using the multi-neuron spike train distance calculated from the vibration data through the neural model were effective in roughness discrimination. These studies suggest the effectiveness of features that take into account the receptive characteristics of mechanoreceptors in texture classification. However, although these studies show high classification accuracy, they use machine leaning models; thus, it is not clear which features contribute to the results and how. Furthermore, we believe that it is necessary to go beyond texture classification and estimate tactile sensation in order to make it useful for product development.

In contrast, as an example of calculating hand-made features from vibration data, Okuyama et al. [26] found a high correlation between the power spectral density of the receptive frequency band of the mechanoreceptor calculated from the vibration data and the evaluation of hair touch feeling. Asaga et al. [27] calculated features from vibration data using a vibration detection threshold of mechanoreceptors and showed that they correlated with tactile factors obtained from sensory evaluation. Although these studies are clear and straightforward about which explanatory variables contribute to tactile sensation, they only employed linear models. In other words, they do not take into account the nonlinearity of the human perceptive mechanism [34,35,36] in the process of tactile estimation. Another previous study has shown that humans perform nonlinear information conversions in two parts of the tactile perception process: one is at the physical interaction between an object and the skin, which has tactile receptors just underneath, and the other is in the brain’s perceptual system where the receptors fire information that is converted into tactile recognition [34]. In particular, neurophysiological studies have revealed that there is no linear relationship between the neural firing from the receptors and the subjective sensation [36], suggesting the importance in performing a nonlinear transformation that takes into account not only the receptive characteristics of mechanoreceptors, but also the brain’s nonlinear perceptual nature. To push the tactile sensing approach forward with the mere basic knowledge to extract feature quantities to a practical level, it is important to show how features can be used in the tactile estimation process, taking into account nonlinearities.

This study aims to show how features based on the characteristics of mechanoreceptors, together with an extension of the feature extraction method, contribute to tactile sensation while introducing nonlinearity into the model by taking into account the nonlinearity of the human tactile perception mechanism.

## 2. Materials and Methods

### 2.1. Strategy of Tactile Estimation Modeling

**Figure 1:**
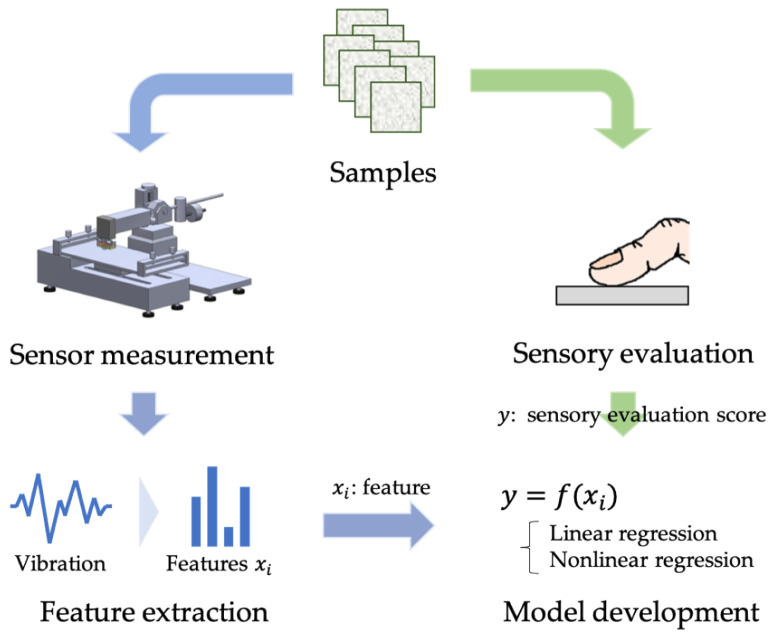
Structure of this study.

The structure of this study is shown in Figure 1 to demonstrate the effectiveness of the nonlinear modeling of tactile estimation. First, we conducted a sensory evaluation test. In parallel, a tactile sensing system was developed, including a newly designed tactile sensor. The vibration data are acquired when a sensor traces a sample, which is followed by feature extraction based on the perceptibility of mechanoreceptors. Then, we developed several model candidates with or without nonlinearities, whose inputs and outputs are the extracted features and sensory evaluation scores, respectively. The mean squared error between the model predictions and the actual values is compared to determine the most effective model.

### 2.2. Target Samples

**Figure 2:**
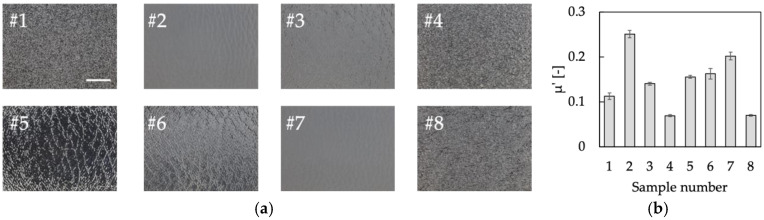
Information of plastic plates. The plates were as follows: #1, polystyrene; #2, unknown; #3, polypropylene; #4, polyethylene; #5, polycarbonate; #6, polymethylmethacrylate; #7, unknown; #8, polyethylene. (**a**) The enlarged views of test samples (scale bar: 5 mm). (**b**) Dynamic friction coefficient, *µ’* (mean ± SD, n = 10).

As shown in Figure 2a, eight plastic plates were selected for target samples as a representative of objects that are widely used in consumer products with textured surfaces. The plates had different surface patterns, which fall within the range of surface roughness of common plastic products. The differences are shown in Table S1 as the arithmetic average roughness of the samples, Ra, and the arithmetic average swell, Wa, measured using the DektakXT (Bruker Corporation, Billerica, MA, USA). The dynamic friction coefficient, *μ’*, was measured using the KES-SE with a 10 mm^2^ piano-wire sensor (Kato Tech Co., Ltd., Kyoto, Japan), as shown in Figure 2b. The total number of samples was determined to be eight so that the subjects could concentrate on evaluating all the samples in an appropriate amount of time.

### 2.3. Sensory Evaluation Test

To quantify the tactile sensations, a sensory evaluation test was conducted with human subjects, performed at a temperature of 24.6 ± 1.0 °C and 41.3 ± 4.5% relative humidity with the participation of 35 healthy adults (21 males and 14 females), with an age of 22.2 ± 1.1 (between 21 and 25) years old. We employed a semantic differential method which is widely used to evaluate the tactile sensation. A seven-step unipolar scale for the Japanese adjectives listed in Table S2 was used as the rating scale. These words were selected by the following procedure. First, we collected 43 Japanese adjectives used for tactile evaluation, referring to previous studies, and narrowed them down to 17 words through brainstorming by the experimenter. Then, to ensure objectivity, a preliminary experiment was conducted in which seven subjects were asked to touch the sample and answer whether or not the words were appropriate for evaluation. As a result, 10 words that more than 70% of the subjects answered as evaluable were used as evaluation words for sensory evaluation. Before the evaluation, the subjects were free to touch all the samples to understand the variety of the samples. During the test, each sample was placed in a box to exclude visual information. The subjects were instructed to touch the sample freely, except for the tracing direction. They were requested to trace the samples only in the horizontal direction with their finger pad. Additionally, in order to avoid the possible order effect, the subjects were allowed to evaluate the samples in a random order, and they were allowed to touch the samples as many times as they wished while scoring an evaluation word. In addition, the evaluation words were given to each participant in a random order to prevent a possible order effect from occurring. The test protocol was approved by the Bioethics Board of the Faculty of Science and Technology, Keio University. The subjects received a thorough explanation of the test methods in advance and then signed an informed consent form before participating in the study. To identify trends in the subjects’ responses, the subjects were classified via cluster analysis using the Python library, SciPy. The scores for all evaluated words were considered in the cluster analysis. The Euclidean distance was used as the distance function, and the Ward method was employed.

For the analysis of tactile sensation, the evaluation words were analyzed by principal component analysis (PCA) for each cluster of subjects based on the evaluation scores using SPSS (Version 22, International Business Machines Corp., Armonk, NY, USA). The conditions for extracting the principal components (PCs) include the criteria that the eigenvalue of each PC should be greater than the unity.

### 2.4. Vibration Measurement System and Procedure

**Figure 3:**
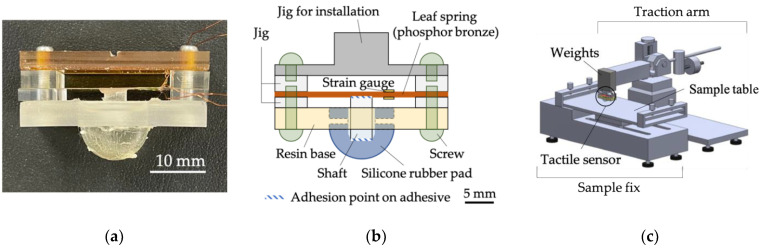
Tactile sensing system with developed tactile sensor. (**a**) Actual image of the tactile sensor. (**b**) Schematic diagram of the tactile sensor structure. (**c**) Overall view of the sensing system.

We developed a tactile sensing system capable of detecting vibrations while a tactile sensor runs over a sample. Vibrations covered here are those caused by a dynamic touch motion, including the stick-slip phenomenon. Figure 3 shows the images of the tactile sensor and an overview of the tactile sensing system. The purpose of this sensor was not to imitate the complex structure of a finger, but to capture the vibrations that occur when the sensor runs over a sample. A cylindrical shaft converts the vertical displacement into the strain of a leaf spring with two strain gauges (KFGS-03-120-C1-23-N30C2, Kyowa Electronic Instruments Co., Ltd., Tokyo, Japan) glued on both sides to detect vibrations when a silicone rubber pad traces a sample surface. The silicone rubber pad was cured by mixing the main compound (SYLGARD^TM^ 184 Silicone Elastomer Base, The Dow Chemical Company, Midland, TX, USA) with the hardener (SYLGARD^TM^ 184 Silicone Elastomer Curing Agent, The Dow Chemical Company, Midland, TX, USA) at 2.5% of the blended amount and a polymerization reaction at 80 °C occurred in air for more than 30 min using a high-temperature dryer. The hardness of the silicone rubber pad was designed to be equivalent to that of a human finger. Young’s modulus of the silicone is reported to be 6.7 MPa, according to the manufacturer. Figure S1 shows a comparison of the hardness between the forefinger pad and the developed sensor measured using a durometer TYPE OO (GS-754G, Teclock Co., Ltd., Nagano, Japan). The results of the Student t-test showed no significant differences between the two. A coating material (X-93-1755-1, Shin-Etsu Chemical Co., Ltd., Tokyo, Japan) was adhered to the surface of the silicone rubber pad. The outputs from the strain gauges were acquired using a dynamic strain amplifier (DPM-913B, Kyowa Electronic Instruments Co., Ltd., Tokyo, Japan). The relationship between the output of the strain gauge, *V,* and the vertical deformation of the tactile sensor, *d,* is shown in Figure S2. From the figure, we can obtain the transformation equation with a linear regression as:

*d* = −1409.4*V* + 710.35. (1)

As shown in Figure 3c, the tactile sensor was fixed to a traction arm of the static and dynamic friction measuring instruments (TL201Ts, Trinity-Lab. Inc., Tokyo, Japan). Upon testing, the normal force *N* between the tactile sensor and the sample can be adjusted by placing weights on the traction arm. As the sample table of the TL201Ts moves horizontally, the tactile sensor runs over a sample. In addition, a force sensor connected to the traction arm detects the tangential force *F*.

The vibration information measurement conditions were as follows: the tracing speed and distance of the tactile sensor were 10 mm/s and 30 mm, respectively. The normal force, *N*, applied between the tactile sensor and sample was 0.49 N. The tracing speed and contact force were determined to be consistent with the general touch conditions reported by previous studies [37,38,39,40]. Measurements were repeated 11 times per sample with a sampling rate of 10 kHz.

### 2.5. Data Processing Methods

There are four mechanoreceptors in the glabrous skin, as shown in Figure S3: Meissner corpuscles (FA I), Pacinian corpuscles (FA II), Merkel disks (SA I), and Ruffini endings (SA II) [31,32,33]. They respond to the mechanical stimuli applied to the skin and then fire nerve impulses to the neuron. The physiological threshold of the amplitude of stimulation for firing against the frequency was reported for each receptor, as summarized in Figure S4. Based on this, we can approximate the threshold line, *L*, on the logarithmic chart for each mechanoreceptor as:

(2) $$L_{\mathrm{FAI}}=\{\begin{array}{l} -17.22\log f+53.91,\;\mathrm{if}\;0.5\;\leq \;f\;\leq \;10.13\\ -12.12\log f+48.78,\;\mathrm{if}\;10.13\;<\;f\;\leq \;14.73\\ 0.2373\log f+34.34,\;\mathrm{if}\;14.73\;<\;f\;\leq \;67 \end{array}$$

(3) $$L_{\mathrm{FAII}}=\{\begin{array}{l} -38.64\log f+64.57,\;\mathrm{if}\;20\;<\;f\;\leq \;237.64\\ 24.93\log f\;-86.48,\;\mathrm{if}\;237.64\;<\;f\;\leq \;800 \end{array}$$

(4) $$L_{\mathrm{SAI}}=\{\begin{array}{l} -10.90\log f+32.77,\;\mathrm{if}\;0.5\;\leq \;f\;\leq \;20.55\\ 9.195\log f+6.390,\;\mathrm{if}\;20.55\;<\;f\;\leq \;120 \end{array}$$

(5) $$L_{\mathrm{SAII}}=\{\begin{array}{l} -17.22\log f+53.90,\;\mathrm{if}\;0.5\;\leq \;f\;\leq \;10.13\\ -12.12\log f+48.78,\;\mathrm{if}\;10.13\;<\;f\;\leq \;128.51\\ -0.6747\log f+24.64,\;\mathrm{if}\;128.51\;<\;f\;\leq \;400 \end{array}$$

where *L*_FAI_, *L*_FAII_, *L*_SAI_, and *L*_SAII_ are the thresholds for FA I, FA II, SA I, and SAII, respectively, and *f* is the frequency of the vibration stimulus. Each mechanoreceptor is supposed to fire when the intensity of the mechanical stimulus surpasses the corresponding threshold line.

**Figure 4:**
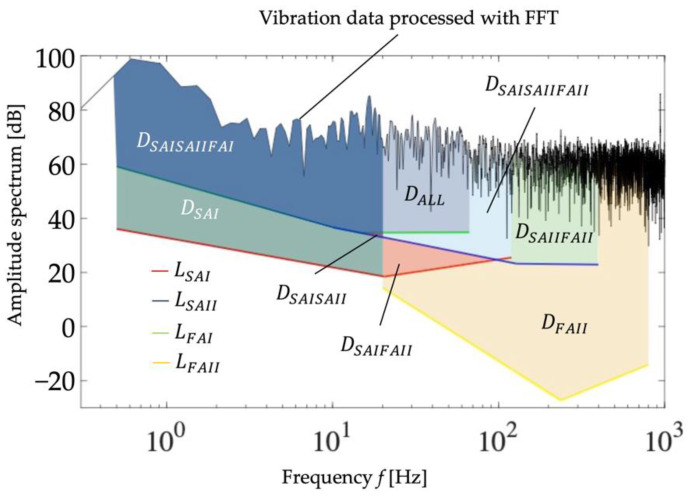
Conceptual diagram for calculation of the features.

The vibration data acquired by the vibration measurement system (Figure 3) were compared with the above-mentioned threshold lines in the frequency domain to extract meaningful information for tactile estimation as follows: First, the acquired output from the strain gauges was converted to the vertical displacement using Equation (1), resulting in the vibration data in the time domain. Then, we transformed the vibration data from the time domain to an amplitude spectrum in the frequency domain using fast Fourier transformation (FFT), implemented in MATLAB (MATLAB 2020a, Math Works Inc., Natick, MA, USA) at a sampling frequency of 10 kHz and mediated with a Hamming window to obtain the vibration data in the frequency domain. The Hamming window was discretized with 32678 points to fit the length of the vibration data. Figure 4 shows a conceptual diagram of the extraction of feature values. The colored area between the lowest threshold and the measured vibration data corresponds to the firing status of the mechanoreceptors. This study considers the combination of firing receptors and divides the entire area into eight subareas, *D_i_*, as shown in Figure 4. The subscript *i* represents the mechanoreceptors that are supposed to fire in the corresponding frequency range. Note that *D_i_* could be zero when the vibration data are always lower than the thresholds. Detailed formulas for calculating each feature are provided in the Supplementary Materials.

### 2.6. Tactile Estimation Models

We performed a regression analysis to predict the principal component scores for each cluster using the features extracted from the vibration data, *D_i_*, and the dynamic friction coefficient, *μ’*, using the Python library and state models. Considering that the human tactile perception nature has nonlinearity [34,35,36], we developed four types of linear/nonlinear regression models:

(6) $$\mathrm{Linear}:\;y=\beta_{0}+\sum_{i=1}^{p}\beta_{i}x_{i}$$

(7) $$\mathrm{Logarithmic}:\;y=\beta_{0}+\sum_{i=1}^{p}\beta_{i}\log\left(x_{i}\right)$$

Interaction: *y* = *β*_0_ + *β*_1_*x_i_*+ *β*_2_*x_j_*+ *β*_1_*x_i_x_j_* (8)

(9) $$\mathrm{Polynomial}:\;y=\beta_{0}+\sum_{i=1}^{a}\beta_{1}x_{1}^{i}$$

where *x_i_* and *x_j_* are the explanatory variables, that is, *D_i_* and *µ’*. *y* is the objective variable, that is, the PC score. *β_i_* represents the coefficients to be determined. In the linear and logarithmic models, regression formulas were constructed for all combinations of variables. *p* means the number of variables to be entered and takes values from one to the maximum number of variables that can be entered. In the interaction model, any two explanatory variables were chosen to build the model, that is, _6_C_2_ = 15 types of models were built for one objective variable. In the polynomial model, only one variable was entered into a single equation. *a* means the maximum number of dimensions of the input variable.

The explanatory variables were introduced using the brute-force method. A regression model was constructed using data obtained from seven out of eight samples. The data for the remaining samples were used to validate the developed regression model. This process was repeated eight times, that is, all samples were used for validation. The model with the lowest error was selected as the best model for each PC in each cluster.

For comparison, we also conducted regression analyses using the feature extraction methods reported in a previous study [27]. In other words, a total of eight regression equations were constructed for one objective value by combining two types of feature extraction methods and four types of models, and the name of each regression model was defined, as shown in Table 1. Previous research [27] only considered three features extracted from the vibration data and used linear regression analysis focusing on the mechanoreceptor with the lowest threshold at each frequency band. Thus, A-1 is the method reported in a previous study [27], and the other models were newly constructed in this study.

## 3. Results

### 3.1. Sensory Evaluation Results

**Figure 5:**
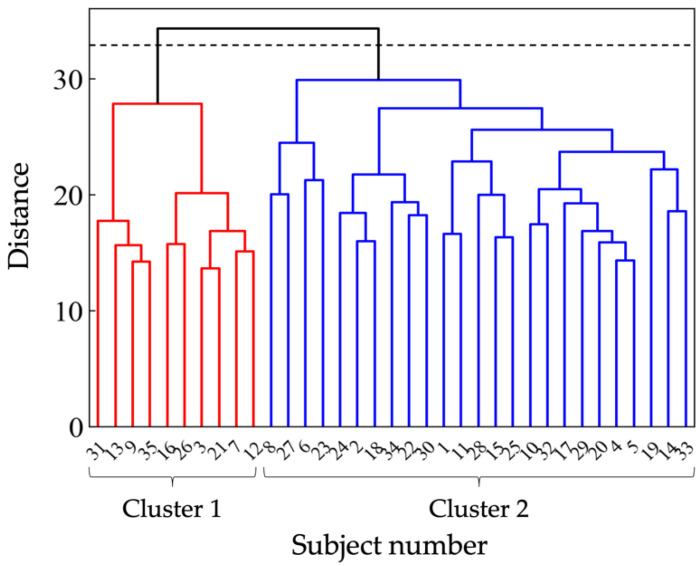
Dendrogram obtained from cluster analysis.

As a result of cluster analysis, the subjects were mainly classified into Cluster 1 (10 subjects) and Cluster 2 (25 subjects), as shown in Figure 5. PCAs were performed on the clusters. The results show two PCs extracted for Cluster 1 and three for Cluster 2, as shown in Table 2. The cumulative contribution rates of the PCA results for Clusters 1 and 2 were 73.2% and 59.7%, respectively. The average PC scores for each sample were considered as objective variables in the following regression analysis.

### 3.2. Feature Values Extracted from Vibration

**Figure 6:**
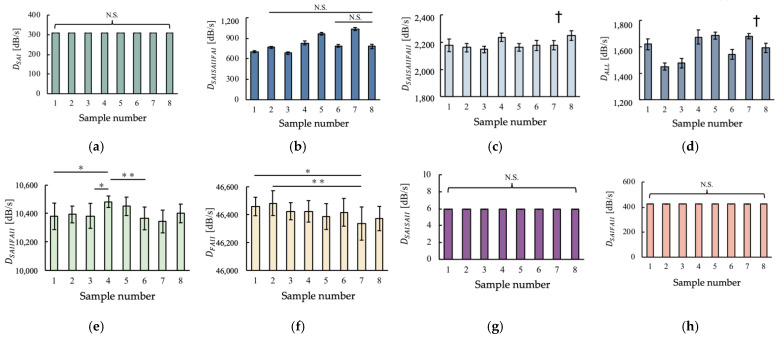
Results of feature calculation for the eight samples. (**a**) *D*_SAI_, (**b**) *D*_SAISAIIFAI_, (**c**) *D*_SAISAIIFAII_, (**d**) *D*_ALL_, (**e**) *D*_SAIIFAII_, (**f**) *D*_FAII_, (**g**) *D*_SAISAII_, (**h**) *D*_SAIFAII_ (mean ± SD, *n* = 11, NS: no significant difference at 5% significance probability, *: *p* < 0.05, **: *p* < 0.01, †: significant differences are noted in the text).

The features corresponding to the eight subareas in Figure 4 were calculated, as shown in Figure 6. As indicated, all the features have different trends among the samples, suggesting that these features extracted from the vibration data could possibly explain the differences in the samples. *D*_SAISAIIFAI_ was significantly different, except between samples (1 and 2, 5, 6, 7), (2 and 3, 5, 6, 7), (3 and 5), (4 and 8), (5 and 6, 7), and (6 and 7). *D*_ALL_ was significantly different, except between samples (1 and 8), (2 and 3), (4 and 5), (4 and 7), and (5 and 7). A one-way analysis of variance showed that there were significant differences (*p* < 0.05) between samples for *D*_SAISAIIFAI_, *D*_ALL_, *D*_SAISAIIFAII_, *D*_FAII_, and *D*_SAIIFAII_. Therefore, these five features for each sample were considered as index variables in the following regression analysis. In addition, the three types of features calculated based on the feature calculation method of a previous study [27] are shown in Figure S5.

### 3.3. Regression Analysis

**Figure 7:**
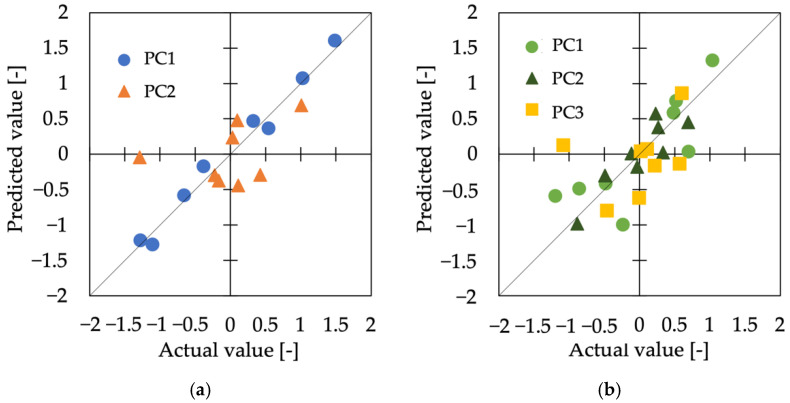
Relationship between the actual value and the predicted value. (**a**) Cluster 1, (**b**) Cluster 2.

Using the features and the dynamic friction coefficient *µ’* as index variables, we performed the regression analysis to estimate the results of the sensory evaluation. The type of model that showed the lowest error for each principal component and its values are shown in Table 3, with the smallest error among all models for each PC shown in bold. The relationship between the values predicted by the model with the smallest error and the measured values is shown in Figure 7.

The regression equations for constructing the model with the smallest error are shown in Equations (10)–(14).

*y_C1PC1_* = −4136 + 5.544log(*D*_SAISAIIFAI_) − 98.20 log(*D*_SAIIFAII_) + 466.3log(*D*_FAII_) + 0.9490 log(*µ*) (10)

*y_C1PC2_* = 1.828 *D*_SAIIFAII_ − 3.5 × 10^−4^ *D^2^*_SAIIFAII_ + 1.69 × 10^−8^
*D*^3^_SAIIFAII_ (11)

*y_C2PC1_* = −16.88 + 2.856 log(*D*_SAISAIIFAI_) + 1.114 log(*µ*) (12)

*y_C2PC2_* = −94.98 + 4.824 × 10^−3^ *D*_SAISAIIFAII_ − 4.270 × 10^−3^
*D*_ALL_ + 8.772 × 10^−3^
*D*_SAIIFAII_ (13)

*y_C2PC3_* = 4911 − 2.237 *D*_SAISAIIFAII_ − 0.4273 *D*_SAIIFAII_ + 2.151 × 10^−4^
*D*_SAISAIIFAI_ *D*_SAIIFAII_ (14)

Note that *y_CiPCj_* is the principal component score of the *j*th PC of Cluster *i*. The coefficient of determination *R*^2^, the adjusted coefficient of determination *R’*^2^, and the *p*-values of each regression equation are shown in Table 4.

## 4. Discussion

As shown in Table 3, the B-*n* models, in which the features were extracted by the proposed method, had the lowest error among all PCs. This implies that considering the combination of firing receptors improves the accuracy of the tactile estimation. As can be seen, the linear regression model (B-1) is effective only for PC2 in Cluster 2, whereas the nonlinear models (B-2, B-3, B-4) are effective for the other PCs. In other words, these results suggest the effectiveness of considering nonlinear models. When the tactile sensation was estimated by the method of a previous study [27], that is, when only the A-1 models were constructed, the mean error of all five PCs was 0.337. In contrast, when both linear and nonlinear models are considered using the features proposed in this study, the average error (for the models shown in bold in Table 3) is 0.190. This is a 43.8% smaller error than that using the A-1 models. These results suggest the effectiveness of building a nonlinear model between the features and the subjective sensory quantities, focusing on nonlinear transformations in the brain from neural firing information to tactile perception. Since the materials used in this study were limited, we believe that we can construct a more versatile model that can estimate a wide range of tactile sensations by building models for different materials and evaluating the differences between them in the future.

Table 4 indicates that the regression equations constructed for PC2 in Cluster 1 and PC3 in Cluster 2 were insignificant, with a significance probability of 5%. In conjunction with the results in Table 3, we can see that the errors of the two models are relatively large (more than 0.3), although they are smaller than those of the previously reported models. Measuring any additional physical quantities may improve the estimation of these tactile sensations.

In the following, we will examine which features effectively explain PC1 in Cluster 1 and PC1/PC2 in Cluster 2, for which statistically significant regression equations were constructed (*p* < 0.05). Table 5 shows the standard regression coefficients, *β’*, and their *p*-values for each variable. PC2 in Cluster 2, where the linear model was effective, had evaluation words with high principal component loadings such as “Slippery” and “Rough”, as shown in Table 2. This implies that the roughness represented by PC2 in Cluster 2 was perceived. Furthermore, we found that the logarithmic model was effective for PC1 in both Cluster 1 and Cluster 2. Both principal components have “Smooth,” “Sticky,” “Pasty,” “Feel friction-drag,” and “Sleek” as the evaluation words with an absolute value of principal component loadings of 0.5 or higher. They are thought to represent similar tactile sensations, such as smoothness. Thus, the results imply that we do not perceive vibration stimuli linearly but logarithmically when perceiving smoothness regardless of the cluster. Contrastingly, PC2 in Cluster 1 and PC3 in Cluster 2, which represent similar tactile sensations, were effectively modeled by different types of models: the interaction model and polynomial model, respectively. This difference may be due to the different perceiving nature of each cluster. Therefore, to make the most of the extracted features in tactile estimation, it is necessary to combine different types of models for different PCs or evaluation words.

## 5. Conclusions

As an alternative to sensory evaluation, estimating human tactile evaluation scores from information acquired by sensors is necessary for the development of products to improve additional values. However, few accurate models for estimating tactile sensation have been reported, and it is necessary to develop an accurate model based on the human tactile perception mechanism. For this purpose, we developed a tactile sensing system capable of detecting vibrations while a sensor runs over a sample. From the vibration obtained, we proposed methods to estimate the firing values of mechanoreceptors focusing on the simultaneous firing of multiple mechanoreceptors. In addition, we conducted sensory evaluations to obtain the sample scores for different evaluation words and extracted principal components for the tactile sensation of samples for the cluster divided by the response tendency. Then, the relationship between the extracted features and tactile evaluation scores was modeled by linear and nonlinear regressions based on the human tactile perception mechanism. The best model was determined by comparing the estimation errors. In conclusion, the results suggest the effectiveness of the feature extraction method proposed in this study and the reduction of error by considering nonlinear models. The results suggest that the proposed nonlinear model improves the average estimation error by 43.8% compared with the previously reported linear model. In addition, the obtained regression equations reveal the physical quantities that contribute to the estimation of smoothness and roughness. In contrast, the *p*-value of some models is not small enough for quantitative tactile estimation. The appropriateness of the conclusion is limited to similar plastic samples since this study only employs plastic plates. As a future task, we expect to improve the tactile estimation by measuring not only vibration, but also other physical properties such as heat flux that may vary depending on different materials.

## Figures and Tables

**Table 1:** Classification of the regression models based upon the feature extraction method and model type.

Model Type	Feature Extraction Method	
	Previously Reported Method [27]	Proposed Method
Linear	A-1	B-1
Logarithmic	A-2	B-2
Interaction	A-3	B-3
Polynomial	A-4	B-4

**Table 2:** The result of principal component analysis. (Bold letters indicate evaluation words with an absolute value of PC loadings of 0.5 or higher).

Evaluation Word	Principal Component Load				
	Cluster 1		Cluster 2		
	PC1	PC2	PC1	PC2	PC3
Smooth	**−0.933**	−0.055	**−0.646**	0.186	**−0.517**
Sticky	**0.913**	0.136	**0.695**	0.300	−0.257
Pasty	**0.872**	0.120	**0.724**	0.385	0.088
Feel friction-drag	**0.877**	0.000	**0.741**	0.220	0.099
Moisten	**0.840**	0.236	0.466	0.371	0.276
Sleek	**−0.845**	0.196	**−0.617**	0.356	−0.033
Slippery	**−0.561**	0.427	−0.200	**0.725**	0.075
Velvety	−0.215	**0.836**	**−0.603**	0.319	0.318
Fine	−0.048	**0.810**	−0.452	0.356	**0.507**
Rough	−0.188	**−0.772**	−0.011	**−0.673**	0.461
Eigenvalue	5.50	2.26	3.18	1.79	1.00
Contribution rates (%)	50.4	22.8	26.3	18.8	14.6
Cumulative contribution rates (%)	50.4	73.2	26.3	45.1	59.7

**Table 3:** The average error of each regression model. (Bold letters indicate the model with the lowest error for each PC).

Cluster	Principal Component	Model							
		A-1	A-2	A-3	A-4	B-1	B-2	B-3	B-4
Cluster 1	PC1	0.134	0.115	0.876	0.138	0.052	**0.018**	0.061	0.876
	PC2	0.539	0.506	1.133	0.795	0.545	0.535	0.451	**0.338**
Cluster 2	PC1	0.328	0.231	0.211	0.426	0.227	**0.209**	0.307	0.542
	PC2	0.268	0.325	0.303	0.733	**0.046**	0.048	0.138	0.321
	PC3	0.418	0.416	0.688	0.360	0.386	0.385	**0.337**	0.441

**Table 4:** Summary of the constructed regression equations.

Cluster	Principal Component	Equation	*R* ^2^	*R’* ^2^	*p*
Cluster 1	PC1	(10)	0.995	0.986	0.000854
	PC2	(11)	0.458	0.241	0.217
Cluster 2	PC1	(12)	0.837	0.772	0.0107
	PC2	(13)	0.935	0.887	0.0077
	PC3	(14)	0.308	−0.211	0.651

**Table 5:** Standard regression coefficients for each variable.

Objective Variable	Explanatory Variable	β′	*p*
*y_C1PC1_*	${\log(D}_{\mathrm{SAISAIIFAI}})$	0.797	0.004
	${\log(D}_{\mathrm{SAIIFAII}})$	−0.426	0.011
	${\log(D}_{\mathrm{FAII}})$	0.469	0.018
	log(μ)	0.438	0.013
*y_C2PC1_*	${\log(D}_{\mathrm{SAISAIIFAI}})$	0.515	0.039
	log(μ)	0.645	0.018
*y_C2PC2_*	${\log(D}_{\mathrm{SAISAIIFAII}})$	0.357	0.072
	$D_{\mathrm{ALL}}$	−0.787	0.005
	$D_{\mathrm{SAIIFAII}}$	0.798	0.005

## Data Availability

The data presented in this study are available on request from the corresponding author.

## Supplementary Materials

The following supporting information can be downloaded at: https://www.mdpi.com/article/10.3390/s22176697/s1, Table S1: Surface properties of the samples (mean, *n* = 7); Table S2: Evaluation terms used for the sensory evaluation; Figure S1: Comparison of the hardness of the fingertip and the tactile sensor; Figure S2: Output voltage of strain gauge against vertical displacement of the sensor; Figure S3: Mechanoreceptors in human finger; Figure S4: Physical threshold-frequency characteristics for mechanoreceptors (data are estimated from [33]); Figure S5: Calculated feature values based on previous method [27], (a) *I_SAI_*, (b) *I_FAII_*, (c) *I_FAI_* (mean ± SD, *n* = 11, NS: no significant difference at 5% significance probability).

## Abbreviations

Abbreviation	Definition
PCA	principal component analysis
PCs	principal components
FFT	fast Fourier-transformation
