# Supplementary material for: Nonlinear Tactile Estimation Model Based on Perceptibility of Mechanoreceptors Improves Quantitative Tactile Sensing
Source: Sensors (Basel). 2022 Sep 4;22(17):6697. doi: 10.3390/s22176697 (PMC9460129; doi:10.3390/s22176697)
Supplement: Supplementary file 1 [file sensors-22-06697-s001.zip › FigureS3_mechanoreceptors.pdf]

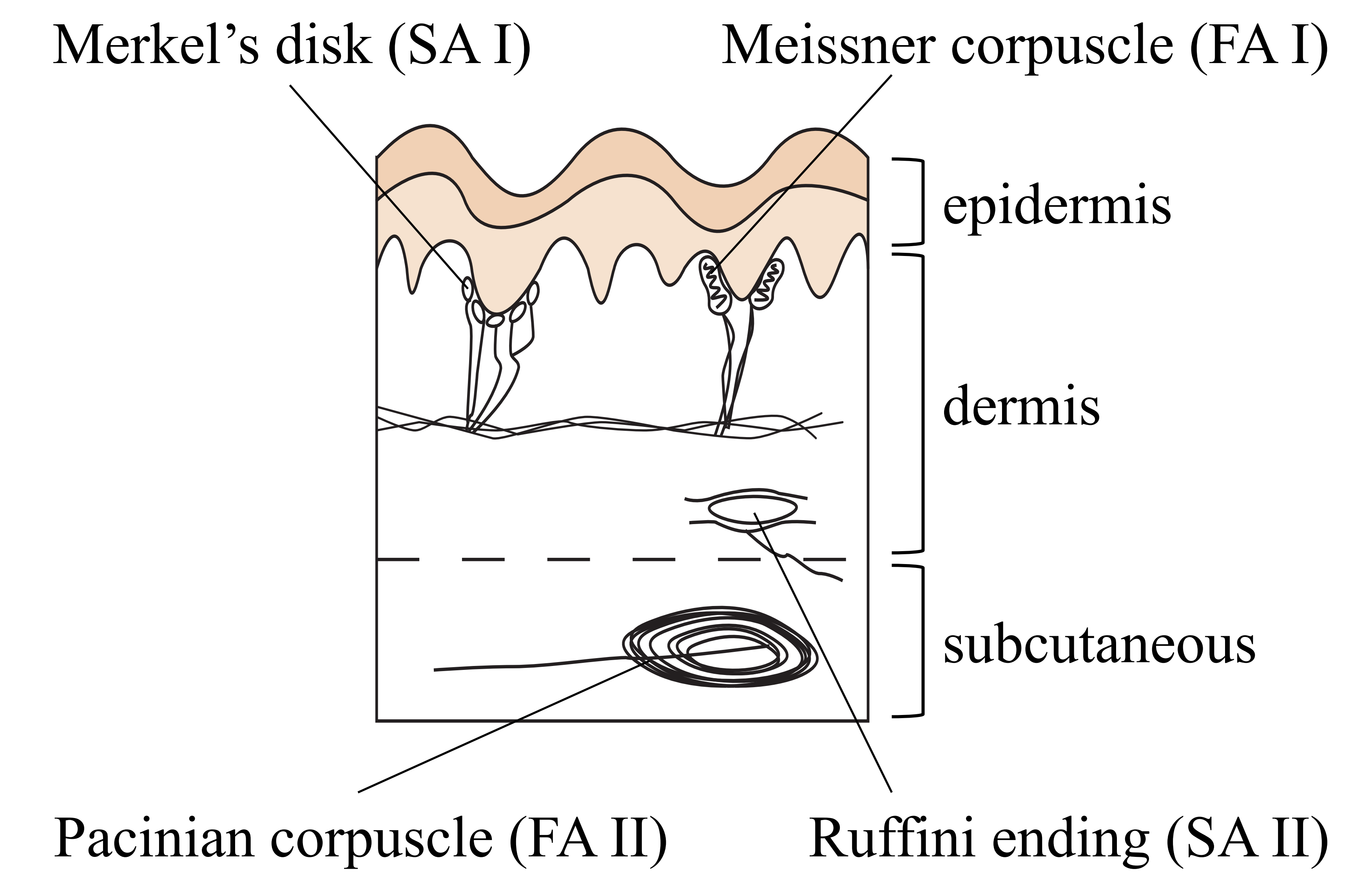
Merkel's disk (SA I)
Meissner corpuscle (FA I)
epidermis
dermis
subcutaneous
Pacinian corpuscle (FA II)
Ruffini ending (SA II)
