# Supplementary material for: Nonlinear Tactile Estimation Model Based on Perceptibility of Mechanoreceptors Improves Quantitative Tactile Sensing
Source: Sensors (Basel). 2022 Sep 4;22(17):6697. doi: 10.3390/s22176697 (PMC9460129; doi:10.3390/s22176697)
Supplement: Supplementary file 1 [file sensors-22-06697-s001.zip › supplement_table.pdf]

Table S1 Surface properties of the samples (mean, n =7)

Sample number	R_a (μm)	W_a (μm)
1	1.699	7.701
2	0.369	3.704
3	0.645	4.228
4	0.576	3.720
5	0.542	4.859
6	0.720	4.373
7	0.280	4.575
8	0.908	6.409

Table S2 Evaluation terms used for the sensory evaluation

Evaluation terms (*Japanese*)			
Smooth (sarasara-suru)	*Sticky (betabeta-suru)*	*Pasty (petapeta-suru)*	*Feel friction-drag (Hikkakaru)*
Sleek (subesube-suru)	*Slippery (tsurutsuru-suru)*	*Velvety (Namerakana)*	*Fine (Kimegakomakai)*
Moisten (Shittori-suru)	*Rough (zarazara-suru)*		

Table S3 Characteristics of subjects in each cluster

Cluster	Male ratio [%]	Percentage of subjects with experience in sensory evaluation [%]
C1	40.0	20.0
C2	68.0	28.0
All subjects	60.0	25.7

The detailed formula for calculation

$$\Delta D_{SAI,i} = \begin{cases} L_{SAII,i} - L_{SAI,i}, & \text{if } L_{SAII,i} < d_i \\ d_i - L_{SAI,i}, & \text{if } L_{SAI,i} < d_i \leq L_{SAII,i} \\ 0, & \text{if } d_i \leq L_{SAI,i} \end{cases} \quad \text{(S1)}$$

$$D_{SAI} = \sum_{i=0.5}^{20} \Delta D_{SAI,i} \tag{S2}$$

$$\Delta D_{SAISAIIFAI,i} = \begin{cases} d_i - L_{FAI,i}, & \text{if } L_{FAI,i} < d_i \\ 0, & \text{if } d_i \leq L_{FAI,i} \end{cases} \tag{S3}$$

$$D_{SAISAIIFAI} = \sum_{i=0.5}^{20} \Delta D_{SAISAIIFAI,i} \tag{S4}$$

$$\Delta D_{SAISAII,i} = \begin{cases} L_{FAI,i} - L_{SAII,i}, & \text{if } L_{FAI,i} < d_i \\ d_i - L_{SAII,i}, & \text{if } L_{SAII,i} < d_i \leq L_{FAI,i} \\ 0, & \text{if } d_i \leq L_{SAII,i} \end{cases} \tag{S5}$$

$$D_{SAISAII} = \sum_{i=14.73}^{20} \Delta D_{SAISAII,i} \tag{S6}$$

$$\Delta D_{ALL,i} = \begin{cases} d_i - L_{FAI,i}, & \text{if } L_{FAI,i} < d_i \\ 0, & \text{if } d_i \leq L_{FAI,i} \end{cases} \tag{S7}$$

$$D_{ALL} = \sum_{i=20}^{67} \Delta D_{ALL,i} \tag{S8}$$

$$\Delta D_{SAISAIIFAII,i} = \begin{cases} \begin{cases} L_{FAI,i} - L_{SAII,i}, & \text{if } L_{FAI,i} < d_i \\ d_i - L_{SAII,i}, & \text{if } L_{SAII,i} < d_i \leq L_{FAI,i} \\ 0, & \text{if } d_i \leq L_{SAII,i} \end{cases} & (20 \leq i < 67) \\ \begin{cases} d_i - L_{SAII,i}, & \text{if } L_{SAII,i} < d_i \\ 0, & \text{if } d_i \leq L_{SAII,i} \end{cases} & (67 \leq i < 97.44) \\ \begin{cases} d_i - L_{SAI,i}, & \text{if } L_{SAI,i} < d_i \\ 0, & \text{if } d_i \leq L_{SAI,i} \end{cases} & (97.44 \leq i \leq 120) \end{cases} \tag{S9}$$

$$D_{SAISAIIFAII} = \sum_{i=20}^{120} \Delta D_{SAISAIIFAII,i} \tag{S10}$$

$$\Delta D_{SAIFAII,i} = \begin{cases} L_{SAII,i} - L_{SAI,i}, & \text{if } L_{SAII,i} < d_i \\ d_i - L_{SAI,i}, & \text{if } L_{SAI,i} < d_i \leq L_{SAII,i} \\ 0, & \text{if } d_i \leq L_{SAI,i} \end{cases} \tag{S11}$$

$$D_{SAIFAII} = \sum_{i=20}^{97.44} \Delta D_{SAIFAII,i} \tag{S12}$$

$$\Delta D_{FAII,i} = \begin{cases} \begin{cases} L_{SAI,i} - L_{FAII,i}, & \text{if } L_{SAI,i} < d_i \\ d_i - L_{FAII,i}, & \text{if } L_{FAII,i} < d_i \leq L_{SAI,i} \\ 0, & \text{if } d_i \leq L_{FAII,i} \end{cases} & (20 \leq i < 97.44) \\ \begin{cases} L_{SAII,i} - L_{FAII,i}, & \text{if } L_{SAII,i} < d_i \\ d_i - L_{FAII,i}, & \text{if } L_{FAII,i} < d_i \leq L_{SAII,i} \\ 0, & \text{if } d_i \leq L_{FAII,i} \end{cases} & (97.44 \leq i < 400) \\ \begin{cases} d_i - L_{FAII,i}, & \text{if } L_{FAII,i} < d_i \\ 0, & \text{if } d_i \leq L_{FAII,i} \end{cases} & (400 \leq i \leq 800) \end{cases} \quad \text{(S13)}$$

$$D_{FAII} = \sum_{i=20}^{800} \Delta D_{FAII,i} \quad \text{(S14)}$$

$$\Delta D_{SAIIFAII,i} = \begin{cases} \begin{cases} L_{SAI,i} - L_{SAII,i}, & \text{if } L_{SAI,i} < d_i \\ d_i - L_{SAII,i}, & \text{if } L_{SAII,i} < d_i \leq L_{SAI,i} \\ 0, & \text{if } d_i \leq L_{SAII,i} \end{cases} & (97.44 < i \leq 120) \\ \begin{cases} d_i - L_{SAII,i}, & \text{if } L_{SAII,i} < d_i \\ 0, & \text{if } d_i \leq L_{SAII,i} \end{cases} & (120 < i \leq 400) \end{cases} \quad \text{(S15)}$$

$$D_{SAIIFAII} = \sum_{i=97.44}^{400} \Delta D_{SAIIFAII,i} \quad \text{(S16)}$$

where, d_i is the value of the amplitude spectrum at *i* Hz, and $L_{m,i}$ is the value of the threshold of the mechanoreceptor *m* at *i* Hz.
