# Supplementary figures and images for: Nonlinear Tactile Estimation Model Based on Perceptibility of Mechanoreceptors Improves Quantitative Tactile Sensing
Source: Sensors (Basel). 2022 Sep 4;22(17):6697. doi: 10.3390/s22176697 (PMC9460129; doi:10.3390/s22176697)

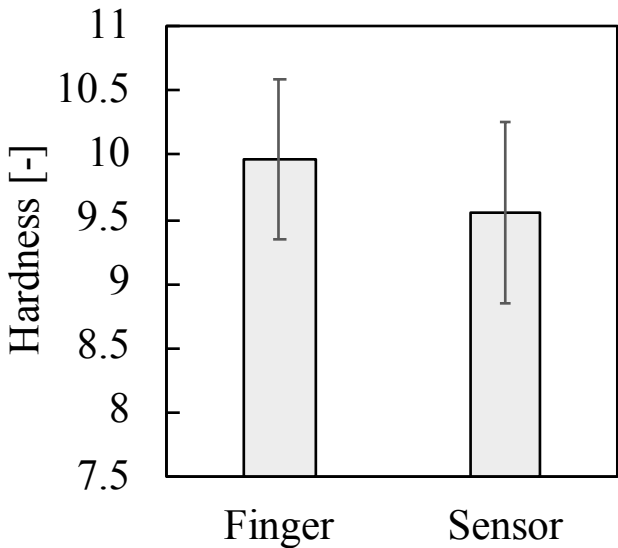

Supplement: Supplementary file 1 [file sensors-22-06697-s001.zip › FigureS1.pdf]

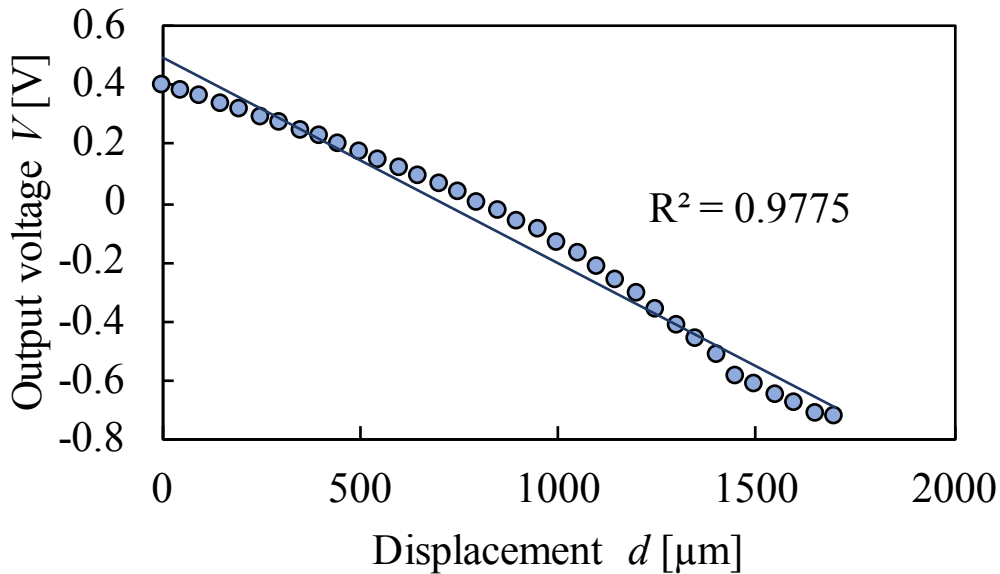

Supplement: Supplementary file 1 [file sensors-22-06697-s001.zip › FigureS2_output_d.pdf]

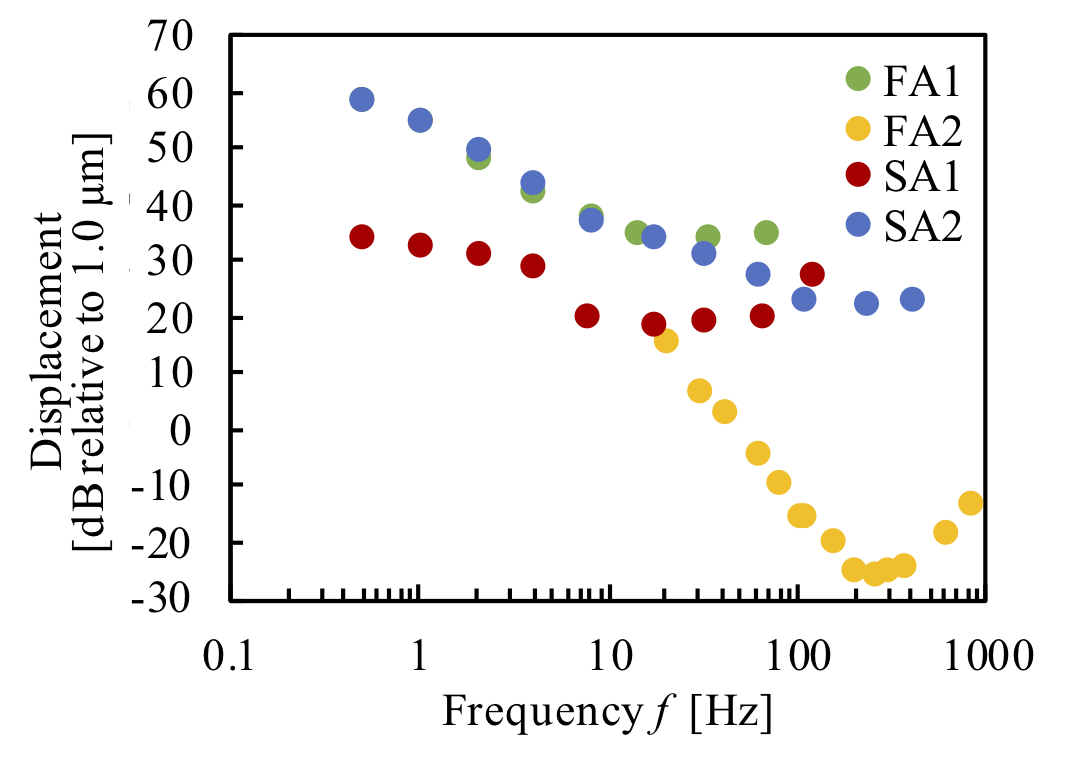

Supplement: Supplementary file 1 [file sensors-22-06697-s001.zip › FIgureS4_mechanoreceptor_threshold.tiff]

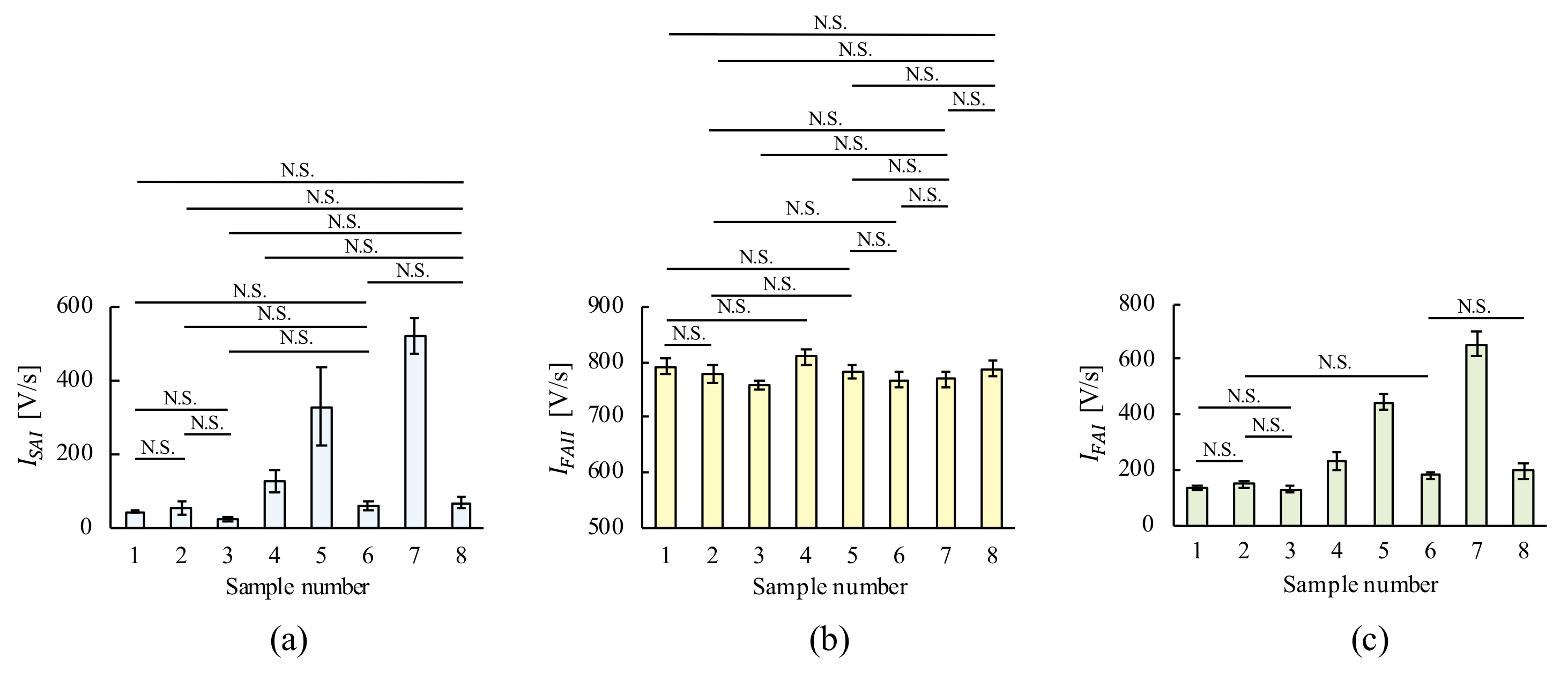

Supplement: Supplementary file 1 [file sensors-22-06697-s001.zip › FigureS5_asaga_features.tiff]
